# Social and Poor-Law Problems

**Published:** 1907-10-12

**Authors:** 


### SOCIAL AND POOR-LAW PROBLEMS.

### A WARNING TO CARELESS PARENTS.

The magistrates of Sedgley have done a thing which is too rarely ventured on; they have fined a man and his wife to the amount of £5 6s. 6d. for letting their children go about while suffering from scarlet fever. One child had gone to school and the other had been out as nurse-girl. The result of this had been to start an epidemic of scarlet fever in the district, and it had been necessary to close two schools. No doubt the parents felt aggrieved by the fine, and might perhaps plead ignorance of the mischief they were likely to cause by their carelessness, but such an exemplary punishment will go far to prevent such ignorance and carelessness in the neighbourhood for a long time. It is a pity that similar action is not taken oftener by the authorities. Many of our epidemics, with all their terrible tale of suffering, death, and sorrow—not to mention the expense to the public—are caused simply by the carelessness and selfishness of a few persons, who, if they go unpunished, do not even perceive the wickedness of their action. A smart punishment is a lesson not only to the offenders but to others, and if such a fine as the Sedgley magistrates inflicted was often demanded from these offenders, it would be better for the health of the nation.

### FREE FIREGUARDS.

The Town Council of Manchester have been interesting themselves in the prevention of accidents to children from fire. At a recent meeting one gentleman moved (1) That having regard to the lamentable loss of life through burning accidents, the Council should urge upon the Government the desirability of legislation to make compulsory the use of fireguards where children are left without adult control; and (2) that the Council should provide them free of cost in all cases where poverty prevented their purchase. The town clerk explained that there is no power to provide fireguards out of the rates, whereupon another member of the Council moved that the Council should recommend "that each and every cottage rented at 5s. a week and under should be provided with a fireguard by the property owner." If such a recommendation should ever be adopted, it is to be hoped that it will be accompanied by some regulations for investigating the circumstances under which the children are left "without adult control," and perhaps also by punishment for mothers who go out on trivial errands or visits to public-houses, when their children get burned. Very few good mothers, even among the poorest, leave their children to run such risks, and though it may be necessary, for the sake of the children, to use public money to protect them against bad ones, the severest means should be employed to instil into the stupid and selfish minds of the latter the fact that they have a personal responsibility for their children's welfare.

### THE NEW PAUPERISM.

After West Ham, Hammersmith! There is no evidence of direct dishonesty in the record of affairs there, but the extravagance in building at least shows woeful indifference to the pockets of the ratepayers. It is true that the outcry about the Hammersmith Workhouse is in some respects unreasonable. An illustrated newspaper shows us a picture of a kitchen fitted with large boilers and roasting-ovens, and of a laundry fitted with steam and electrical machinery. But there is no inevitable extravagance in the existence of these. Wherever hundreds of people have to be catered for there must be machinery, the initial cost of which is great, to do the work, whether the guests be millionaires or paupers. It is more than doubtful if coal fires and wooden wash-tubs in sufficient numbers would prove much cheaper. Even the covered way for the paupers is not in itself a wrong to the ratepayer. A large proportion of workhouse inmates are old and feeble. It would be inhuman to make them go from one part of the institution to another exposed to every change of our variable climate, without shelter of any kind, and the very people who are now crying out against this comfort would be the first to echo a cry of cruelty if it were missing. We must accept the fact that one cannot build even a workhouse for nothing. But when it is built in any but the plainest fashion, extravagance—unjustifiable extravagance—begins, and to this Hammersmith must plead guilty. Whether or not there is worse behind we are not yet in a position to say. But whether the fault is mere carelessness, or, as in West Ham, bribery and theft, the primary fault lies with the ratepayers themselves. It is the easiest thing in the world to become a guardian. So little interest is taken in the election of them that almost any one may be elected without a contest, almost without a question. Even if there is a contest it is as likely as not to be fought on grounds of politics which have nothing whatever to do with Poor-law administration. And from the ratepayer's point of view there is not much to choose between the guardian who steals their money for himself and his friends, and the one who lavishes it on the poor without respect to their deserts, and makes the pauper better off than many of those who support him. Even when it is not ratepayers' money that pays for them, it is easy to go too far in the providing of comforts for paupers. Most people will agree with Mr. Eliot that even though they were private persons who gave billiard-tables for the men at the Hollesley Bay labour colony, the gift was a mistake, and that the men were pampered. Mr. Lansbury blamed the Local Government Board and Mr. John Burns because money was not forthcoming from the national exchequer to set the members of the colony up as small landholders. We are of opinion that the refusal shows sound wisdom. The training of the colony, with its assured comforts and absence of responsibility was by no means fitted to make successful small farmers, who of all men must work late and early, "shun delights and lead laborious days, if ever they are to turn sand into gold." Let the colonists go first as ordinary agricultural labourers, and work and live under the ordinary conditions of such, before they demand land of their own. A year or two of that training will make them far more competent to manage a holding—and perhaps less eager. It can hardly be denied that their life at the colony was far easier than an ordinary labourer's. Indeed, the whole tendency of Poor-law management in recent years has been to make the pauper's life easier than that of the poorer sort of independent men. One result of this is to be found in the readiness with which able-bodied men accept the shelter of the workhouse. One of the Edmonton guardians remarked recently that in that workhouse there were many able-bodied, capable mechanics who preferred to remain there in idleness, a burden on the ratepayers, rather than work for less than the wages fixed by their trade-unions. Perhaps their fellow-unionists regard this as a noble self-sacrifice on their part, but the ratepayer who has to support them will think otherwise. One man who had been in the Edmonton workhouse was allowed to take his discharge on condition that he contributed the sum of 6s. a week towards the support of his four children! And he had paid only one contribution, pleading that his work was irregular. As, however, he had been in prison in the interval there might be sufficient reason for his being out of work. Without assuming that this man was a fair sample of the average pauper, one is justified in saying that ill-advised leniency in dealing with such characters or with those who are simply lazy is an injustice to decent people. They cannot be refused the shelter of the workhouse, but there should be such differentiation among the inmates as would make life there fairly hard to the ablebodied, without involving harshness to the aged and the infirm.

